# Prevalence and Selected Sociodemographic of Movement Behaviors in Schoolchildren from Low- and Middle-Income Families in Nanjing, China: A Cross-Sectional Questionnaire Survey

**DOI:** 10.3390/children7020013

**Published:** 2020-02-13

**Authors:** Si-Tong Chen, Jin Yan

**Affiliations:** 1 Department of Physical Education, Shenzhen University, Shenzhen 518060, China; 2 School of Humanity and Physical Education, Nanjing Sport Institute, Nanjing 210014, China; 3 Faculty of Education and Arts, University of Newcastle, Callaghan, NSW 2308, Australia; jin.yan@uon.edu.au

**Keywords:** 24-h movement guidelines, physical activity, screen time, sleep, youth, low and middle income, China

## Abstract

Background: To investigate the prevalence of meeting the 24-h movement guidelines and its sociodemographic correlates in youth from low- and middle-income families (LMIFs) in Nanjing, China. Methods: Cross-sectional data on moderate to vigorous physical activity (MVPA), screen time (ST), and sleep (SLP) were collected using the Chinese version of Health Behavior School-Aged Children questionnaire among grade schoolchildren of 4th–12th (mean age 13.6 years). The prevalence of meeting the 24-h movement guidelines was in line with the recommendations of the Canadian 24-h movement guidelines. Generalized linear models were used to explore the relationships of correlates with the prevalence of meeting the movement guidelines. Results: The prevalence of meeting the MVPA, ST, and SLP guidelines and their combination was 9.9%, 65.2%, 37.2%, and 2.9%, respectively. As for the prevalence of meeting the MVPA guidelines, boys, younger schoolchildren, and those living in urban areas had a higher chance of meeting them. With regards to the prevalence of meeting the ST guidelines, girls, urban schoolchildren, and the oldest participants were more likely to meet the guidelines. Residential areas and grades were two correlates of meeting the SLP guidelines as well as 24-h movement guidelines. Conclusions: The majority of schoolchildren from LMIFs in Nanjing, China had unhealthy movement behaviors. This concerning situation was mainly predicted by schoolchildren’s grades, indicating older schoolchildren exhibited lower levels of movement behavior. Improved studies are encouraged to identify the correlates of movement behaviors in schoolchildren, which in turn designs and implements effective interventions.

## 1. Introduction

Studies have demonstrated that physical activity (PA) [1,2], screen time (ST) [3,4], and sleep (SLP) [5] are linked with health outcomes in young people. In particular, recent studies indicated that having sufficient PA (moderate to vigorous PA, or MVPA), limited ST, and adequate SLP duration concurrently leads to more desirable health outcomes than the presence of any single healthy behavior in school-aged children [6,7]. Thus, finding ways to improve MVPA and SLP while limiting ST is an important research issue in the public health field [8], which has provoked countries, such as Canada [9], Australia [10], and New Zealand [11], to develop 24-h movement guidelines. Those guidelines commonly recommend that young people should accumulate 60 min of MVPA, have no more than 2 h of ST, and amass 8–10 h of SLP per day [9].

Using the 24-h movement guidelines as a research paradigm [8,11], a number of studies have examined the rates of young people meeting the guidelines. Lee et al. [12] found that only 1.6% of children and young people aged 12 to 17 years old in South Korea met the movement guidelines while an international collaborative study by Roman-Viñas et al. [7] reported that only 7.2% of 9- to 11-year-old children from 12 countries met the movement guidelines. In the latter study, the rates of meeting the guidelines ranged from 14.9% (Australia) to 1.5% (China), indicating large variations between different countries or regions [7]. A recently published study including rural participants also observed that schoolchildren in rural areas (17.7%) showed higher rates of meeting movement guidelines than those in urban areas (3.6%) [13]. To the authors′ knowledge, the current study is the first to investigate compliance rates for movement guidelines in schoolchildren from socio-economically disadvantaged regions (low- to middle-income countries, or LMICs), as previous studies have generally concentrated on upper-middle- or high-income countries [13], thus limiting researchers’ understanding of specific populations’ movement behavior patterns. Such limitations offer a barrier to healthy lifestyle promotion. Overall, most results suggest a relatively low level of healthy movement behaviors; however, most studies have lacked an investigation of movement behaviors in children and adolescents from low-income families, whether by nation or region, thus limiting the overall findings.

To promote healthy lifestyles among schoolchildren, a better understanding of the correlates and determinants of movement behaviors is crucial, as understanding these factors is more likely to promote effective involvement and lead to improvements in movement behaviors [11]. Previous research has reviewed the correlates of movement behaviors among schoolchildren from various countries [13,14,15]: A cross-sectional study by Manyanga et al. [13] suggested that only outdoor time was a significant correlate of adherence to 24-h movement guidelines while another study indicated that age, siblings, and parents were socio-demographic correlates linked with movement behaviors in children aged 5 to 17 years old [14]. Despite some factors being demonstrably correlates of movement behaviors, the key factors affecting schoolchildren in low-income families remain unclear. In turn, this impedes an understanding of the correlates of movement behaviors generally. From the perspective of health equity [16], learning about socio-economically disadvantaged schoolchildren is necessary to promote population-level health; thus, research on movement behaviors in schoolchildren from low-income families should be given much greater attention in the future.

Over the past decades, faced with the health burden of insufficient PA, prolonged ST, and inadequate SLP in schoolchildren, the Chinese government has issued many policies to resolve the health problem [17,18]. However, there is a lack of evidence-based information on how to design policies and interventions to resolve these health problems. The most recent studies stress the importance of the combination of PA, ST, and SLP [8,11] rather than focusing on single movement behaviors. However, little is known about the levels of all movement behaviors in Chinese schoolchildren generally, and this is especially true of those from low- and middle-income families.

Thus, this study aimed to investigate the levels of all target movement behaviors to investigate the frequency of meeting 24-h movement guidelines among schoolchildren from low- and middle-income families in Nanjing, China. It then explored selected correlates, including gender, grade, and parental education level, based on their popularity within the existing literature.

## 2. Materials and Methods

### 2.1. Study Design and Participants

This cross-sectional study took place from November to December in 2019. Using a multistage sampling technique, public schools in four districts in Nanjing were recruited. The sampling procedure involved sampling districts, towns, and local community districts to develop a representative mix of rural and urban areas; the smallest sampling unit was primary (Grades 4–6), middle (Grades 7–9), and high schools (Grades 10–12). Students’ sampling took place in classes selected randomly from each grade in the selected schools.

This study excluded children under 10 years old (1st–3rd graders) owing to their limited ability to respond to surveys. The study recruited 7356 students in total, with responses received from 6788 participants (92.3% response rate) who completed the questionnaire survey. The Institutional Review Board of Nanjing Sport Institute approved the study protocol, and the teachers and principals of the participating schools assisted in obtaining permission to conduct the study. All the children and adolescents participating in the study, along with their parents or guardians, were informed that participation was voluntary. Each student and their parents were informed of the ethical issues during data collection by the instructions on the frontpage of the questionnaires. If students and their parents returned the questionnaires, they were regarded as providing informed consent to participate in the study. Data were collected and analyzed anonymously.

### 2.2. Procedures

All selected participating children and adolescents were informed about the survey before data collection. Participants received detailed directions on how to answer the survey. Trained research assistants conducted a study of physical activity and assessment of body weight and height during regular school time.

### 2.3. Measures

#### 2.3.1. Demographics

Participants were asked for demographic information by means of a self-reported questionnaire. Data included sex (boy or girl), grade (4 to 12), race (Han Chinese or other), and type of living area (urban or rural). Socioeconomic status (SES) parameters included parental educational status for both parents (less than college or university; college or university; postgraduate) and family affluence based on per capita annual income (RMB: <9000; 9000–30,000; >30,000–100,000; >100,000), and these were collected using a parent-reported questionnaire based on Cirino et al. [19]. Based on previous measures of family income and Chinese society’s economic development, low- and middle-income families were defined as those with per person annual incomes of no more than RMB 30,000.

#### 2.3.2. Movement Behaviors

Two items of the Health Behavior in School-Aged Children (HBSC) questionnaire measured participants’ PA [20], including: (1) How many days did you participate in MVPA for at least one hour on weekdays in the past week? (0 = none, 1 = 1 day, 2 = 2 days, 3 = 3 days, 4 = 4 days, 5 = 5 days); (2) How many days did you participate in MVPA for at least one hour on the weekend in the past week? (0 = none, 1 = 1 day, 2 = 2 days). To help participants better understand MVPA, it was explained as, “Any kind of physical activity that increased your heart rate and made you breathe hard some of the time (including physical education time, exercise, sports training, and various regular daily activities, such as brisk walking, hiking, and excursion).” The overall MVPA level was calculated using the following formula: Overall MVPA = MVPA days on weekdays + MVPA days on weekend. According to the 24-h movement guidelines [9], meeting the MVPA recommendation is defined as reporting seven days with a minimum of 60 min of MVPA daily.

ST measurements used the following reliable and valid items derived from the HBSC [20]: (1) How many hours did you spend watching TV or movies in your leisure time on weekdays and weekend days over the past week, respectively? (2) How many hours did you spend playing video games in your leisure time on weekdays and weekend days over the past week, respectively? (3) How many hours did you spend in activities using electronic screen-based devices in leisure time on weekdays and weekend days over the past week, respectively? The responses to these questions were none, about a half-hour, 1 h, 2 h, or 3 h or more. The average daily ST hours was calculated using the following formula: average daily ST hours = (ST hours on weekdays × 5 + ST hours on weekend × 2)/7. According to the Canadian 24-h movement guidelines [9], meeting the ST recommendation required daily ST was ≤2 h per day.

SLP was measured by the items derived from the HBSC questionnaire [20]: (1) When do you usually go to bed if you have to go to school in the next morning? (2) When do you usually go to bed at weekends or during holidays? (3) When do you usually wake up on school mornings? (4) When do you usually wake up at weekends? The answers to items 1–4 were as follows: (1) No later than 21:00; 21:30; 22:00; 22:30; 23:00; 23:30; 24:00; 00:30; 01:00; 01:30; 02:00 or later; (2) no later than 21:00; 21:30; 22:00; 22:30; 23:00; 23:30; 24:00; 00:30; 01:00; 01:30; 02:00; 02:30; 03:00; 03:30; 04:00 or later; (3) no later than 05:00; 05:30; 06:00; 06:30; 07:00; 07:30; 08:00 or later; and (4) no later than 07:00; 07:30; 08:00; 08:30; 09:00; 09:30; 10:00; 10:30; 11:00; 11:30; 12:00; 12:30; 13:00; 13:30; 14:00 or later. Participants’ response was used to calculate the SLP duration of the night on weekdays and the weekend, respectively. Then, the average SLP duration per night (hours) was calculated using the following formula: average SLP duration = (SLP duration per night on weekdays × 5 + SLP duration per night on weekends × 2)/7. Based on the 24-h movement guidelines [9], 9–11 h and 8–10 h are recommended for 6–13-year-olds and 14–17-year-olds, respectively.

#### 2.3.3. Weight Status

Body mass index (BMI), as calculated by the weight to height ratio, was used to determine participants′ weight status, and well-trained research staff followed a standardized protocol to acquire these measurements. Weight was measured to the nearest 0.10 kg with a balance-beam scale while the participants were wearing lightweight clothing, and height was measured to the nearest 0.10 cm with a portable stadiometer while participants were barefoot. Hand-held instruments were used to assess both measures. BMI values were calculated as (kg/m^2^). The sex- and age-specific cut-offs for overweight (OW) and obese (OB) categories as established by the World Health Organization (WHO) [21] were used to divide participants into non-OW/OB and OW/OB groups.

#### 2.3.4. Statistical Analysis

The current study focused on participants from low- and middle-income families, thus we deleted cases of participants from upper-middle- and high-income families (*n* = 3576). After removing invalid and abnormal values from all independent and dependent variables of the eligible participants (*n* = 598), the final sample size was 2614 responses. IBM SPSS 24.0 was used to perform all statistical analyses. Descriptive statistics were used to examine the characteristics of samples, the prevalence of meeting each recommendation in isolation and the prevalence of combinations, and the prevalence of non-OW/OB and OW/OB characteristics. After confirmation of the data distribution was achieved, a Chi-Square test was applied to examine sociodemographic differences in MVPA, ST, and SLP levels. Generalized linear models (GLMs) were then used to investigate the associations between the prevalence of meeting the recommendations (in isolation or combination) and weight status. These models set study sites (districts) as fixed effects and schools as random effects. The level of statistical significance was set at *p* < 0.05 for all tests

## 3. Results

The demographic characteristics of the sample are shown in Table 1. The sample consisted of 2614 participants. Just over half the sample were girls (50.8%), with the mean age of children and adolescents being 13.6 years old (±2.6). Among the sample, primary (grade 4–6), secondary (grade 7–9), and high school (grade 10–12) students accounted for 33.6%, 30.8%, and 35.6%, respectively. Additionally, more than half of the sample live in urban areas (51.8%), are from a single child family (51.7%), and are from low- or middle-monthly family-income (59.6%). Only 9.3% of children had parents who were at college, university, or other higher educational levels. In terms of BMI percentile, 15.8% and 6.8% of youth were classified as overweight and obese, respectively.

Table 2 shows the percentage of children and adolescents meeting the MVPA, ST, SLP, and 24-h guidelines with the prevalence of each demographic characteristic group. Overall, the percentages of youth who met the MVPA, ST, SLP, and 24-h guidelines were 9.9%, 65.2%, 37.2%, and 2.9%, respectively. The percentage of boys that met the three guidelines was greater than those of girls (MVPA: 11.6% > 8.4%, SLP: 37.9% > 36.6% and 24-h: 3.2% > 2.6%), except for the ST guideline (ST: 62.3% < 68.1%). Among the children who live in urban areas, the percentage of children achieving the MVPA, ST, and 24-h guidelines was higher than those in rural areas (MVPA: 12.4% > 7.4%, ST: 68.7% > 61.5% and 24-h: 4.0% > 1.7%). However, this was not the case with meeting the SLP guideline (SLP: 35.0% < 39.6%). According to the grade groups, the percentage of participants complying with the MVPA, SLP, and 24-h guidelines decreases as the grades increase (MVPA: 12.8% > 9.5% > 7.6%, SLP: 77.1% > 25.4% > 9.9% and 24-h: 5.9% > 2.2% > 0.6%. However, the situation was not the same for the ST guideline (grade 4–6: 62.1%, grade 7–9: 60.6%, grade 10–12: 72.2%). Children and adolescents who have no siblings were more likely to meet the MVPA and ST guidelines but less likely to meet the SLP and 24-h guidelines. With participants from low- and middle-income families, youth were more likely to meet the ST, SLP, and 24-h guidelines. With regards to the parental educational levels, the percentage of participants meeting the MVPA guideline was the same. Among those whose parents possess a college, university, or other higher education level, the percentage of participants meeting the ST and 24-h guidelines was also higher. There was a decreasing trend among the percentages of participants meeting the ST guideline (normal: 66.4%, overweight: 61.8%, obesity: 60.1%), whereas there was an increasing trend among the percentage of participants meeting the SLP and 24-h guidelines (normal: 35.8%, overweight: 40.3%, obesity: 46.3% for SLP guideline; normal: 2.8%, overweight: 2.9%, obesity: 3.9% for 24-h guideline).

Table 3 shows the associations between the MVPA, ST, SLP, and 24-h guidelines with each demographic characteristic. Specifically, boys were 1.5 times more likely to meet the MVPA guidelines compared with girls (OR = 1.5, 95% CI: 1.1–1.9). However, girls had a 30% greater chance of meeting the ST guidelines compared with boys (OR = 1.3, 95% CI: 1.1–1.5). A non-significant association was discovered between the SLP and 24-h guidelines with gender. With regards to living areas, children and adolescents living in urban areas had a higher likelihood of complying with both the MVPA and ST guidelines (OR = 1.8, 95% CI: 1.4–2.4; OR = 1.4, 95% CI: 1.2–1.7). On the contrary, youth who lived in rural areas were more likely to achieve both the SLP and 24-h guidelines (OR = 1.3, 95% CI: 1.1–1.6; OR = 2.5, 95% CI: 1.5–4.1). Compared with older adolescents in grade 10-12, adolescents in grade 7-9 were 1.1 (OR = 1.1, 95% CI: 0.8–1.6), 3.3 (OR = 3.3, 95% CI: 2.5–4.3) and 2.8 (OR = 2.8, 95% CI: 1.1–7.3) times more likely to meet the MVPA, SLP, and 24-h guidelines, respectively. Younger children in grade 4–6 were 1.7 (OR = 1.7, 95% CI: 1.3–2.4), 31.2 (OR = 31.2, 95% CI: 23.8–40.8), and 9.3 (OR = 9.3, 95% CI: 4.0–21.9) times more likely to meet the recommendations for the MVPA, SLP, and 24-h guidelines. As for the ST guidelines, compared to younger children in grades 4–6, elder adolescents in grades 10-12 were 1.6 (OR = 1.6, 95% CI: 1.3–2.0) times more likely to meet the recommendations. However, adolescents in grades 7–9 were 0.9 (OR = 0.9, 95% CI: 0.9–1.1) times less likely to meet it. Additionally, children whose parents had attained a college, university, or higher-level education were 1.7 (OR = 1.7, 95% CI: 1.3–2.4) times more likely to comply with the ST guideline. Nonetheless, non-significant associations were discovered in the MVPA, SLP, and 24-h guidelines with parental educational levels. Similar situations were discovered in terms of family composition, family income, and weight status, and a non-significant association was revealed for the four guidelines mentioned above.

## 4. Discussion

Few studies, such as the present study, have investigated the prevalence of meeting the 24-h movement guidelines and its potential correlation with schoolchildren from low- and middle-income families in Nanjing, China. This study found that only 3 out of 100 children met the 24-h movement guidelines, and that this prevalence was correlated with both residential areas and grades. With regards to the prevalence of meeting the MVPA, ST, and SLP recommendations, a large variation was observed, with each being explained by different predictors.

The present study indicates that those from low- and middle-income families exhibit a low prevalence of healthy movement behaviors. Compared with previous studies containing participants from high-income families [7,12], a lower level of participants who met the 24-h movement guidelines was observed. For example, an international study that included 12 nations found that 7.2% of children aged 9-11 years met the 24-h movement guideline [7], and Lee et al. [12] found that 3.2% of young people in South Korea met the movement guidelines; these rates are higher than those observed in this study, which implies that schoolchildren from low- and middle-income families exhibit a concerning lack of healthy movement behaviors. There is evidence that having improved movement behaviors can lead to more desirable health outcomes [6]; thus, the promotion of healthy movement behaviors is vital. In addition, from the perspective of health promotion equity [16,22,23], schoolchildren from low- and middle-income families should be regarded as an interventional priority with regard to improving healthy movement behaviors based on the observed discrepancy.

In seeking to better understand the patterns of movement behavior in schoolchildren, this study found that both residential areas and grades were correlated with meeting movement behaviors. However, little is known about the correlation with movement behaviors. Across a number of limited studies, more outdoor time [13], being younger [14], and family composition (more numbers of children) [14] were seen to be correlated with improved movement behaviors, while in the current study, residential areas and grades were found to be predictors of movement behaviors, suggesting that schoolchildren dwelling in urban areas and with lower grades are more likely to meet the movement guidelines. Consistent with previous studies [12], increasing grades or older children exhibited a lower prevalence of movement behaviors. This study confirms this finding, with the possible reason being increasingly heavy academic pressure. Owing to the academic pressure [24], older schoolchildren have less time for engagement in PA and sleep whilst spending more ST studying. Similarly, a study by Zhu et al. [25] found that children and adolescents who did not report experiencing academic pressures were more likely to meet the PA guidelines (OR = 5.38). This strongly suggests that older schoolchildren are exposed to higher risk behaviors, which may result in a lower prevalence of meeting the movement guidelines. In line with Manyanga et al. [13], the current study also found that residential area is a predictor of movement behaviors: Schoolchildren living in urban areas exhibited a higher prevalence of meeting the MVPA and ST guidelines, which to some extent are major drivers of better movement behaviors. Schoolchildren dwelling in urban areas often have access to additional sports facilities or equipment [26], which may increase their MVPA levels. Additionally, schoolchildren who live in urban areas were more likely to spend more time in non-screen based sedentary time, such as doing homework and reading [24,27], which may decrease screen-based time. Collectively, these factors may explain why schoolchildren from urban areas exhibited higher levels of movement behaviors. However, owing to the limited amount of available evidence, more studies are required to explore the correlations between movement behaviors and schoolchildren′s economic family status.

In this study, the prevalence of meeting the MVPA guideline is relatively low, which is lower than that in other studies [24,28,29]. Schoolchildren from low- and middle-income families are in disadvantaged circumstances regarding engaging in PA because of less resources for PA (e.g., sports facilities and equipment). In addition; relative to those with a higher family income, schoolchildren from low- and middle-income families often lack parental support and accompaniment for PA [30], which in turn results in lower levels of MVPA. Therefore, these groups need more interventional targeting. Considering the time of data collection (winter) for this study, seasonality may also be a possible reason for the low MVPA prevalence, as previous studies have shown that children and adolescents exhibited lower levels of MVPA in winter as compared with other seasons [31,32,33]. However, there is minimal evidence to support the effects of seasonality on PA in Chinese children and adolescents; thus, future studies should address this research gap. Unlike previous studies, this study did not find any significant relationships with family composition, family income, parental education level, or weight status and MVPA. However, more studies in Chinese populations are encouraged to explore these factors for more subtle correlations with MVPA.

This study found that over 60% of schoolchildren met ST guidelines, which is an acceptable level that is consistent with other studies [24,26,27]. It is commonly recognized that excessive ST is detrimental for the health of young people [3,34]; however, although the majority of schoolchildren met ST guidelines, more efforts must be made to reduce the percentage of schoolchildren who have more than 2 h of ST per day. This study also indicated that female and urban schoolchildren had more optimal levels of ST, consistent with previous studies in Chinese children [24,26]. Higher parental educational level also appeared to predict less ST across previous studies [27], and this study confirms this finding; this may be explained by better parental role modelling encouraging schoolchildren to spend less time in front of a screen. Surprisingly, however, this study found that students with higher grades were more likely to meet ST guidelines, a result inconsistent with previous studies [24,35]. This interesting discovery may be interpreted as showing that students with higher grades take on more homework, which thus occupies more of their time, reducing the time available for screen-based activities. However, as little other research has produced such findings, this explanation is offered only as a possibility.

SLP in schoolchildren is of particular interest for the current research and for practice in public health due to its positive relationships with more desirable health outcomes [5]. More than 35% of schoolchildren in this study met the SLP guidelines. Compared with previous studies [7], the results thus show a higher general level of SLP. Such inconsistencies could be the result of different measures being used. In addition, SLP in Chinese schoolchildren has been relatively understudied. Future studies should use comparable data to monitor SLP levels among Chinese schoolchildren to help promote schoolchildren obtaining appropriate sleep durations on a regular basis. This study found that the frequency of meeting SLP guidelines declined significantly with increases in grades, which can be explained by heavier academic pressure [36]. Increasing grades, regardless of family income, indicate that schoolchildren face excessive academic loads, with consequent decreases in their SLP durations. This study also found that schoolchildren living in rural areas had a higher likelihood of meeting SLP guidelines, though previous studies have not indicated this, limiting support for this finding. A possible reason is that schoolchildren living in rural areas are exposed to less ST at night, which may support higher rates of meeting SLP guidelines. A possible reason is that schoolchildren living in rural areas were exposed to less time spent in ST at night, which may support the higher prevalence of meeting the SLP guidelines.

This study has several strengths. Firstly, the present study is one of very few investigations examining movement behaviors in Chinese schoolchildren, and thus it may help advance knowledge in this field by providing further guidance and implications for future studies and practice. In addition, compared with previous studies, this study accessed a relatively large sample, enlarging the study findings by widening the demographic range involved. This study also adopted widely accepted measures to allow comparison of the results with other studies. However, there are some limitations, which also need to be mentioned. One major concern regarding this study is the use of subjective measures to collect data on movement behaviors, which may result in bias in estimating the prevalence of meeting the guidelines. Another limitation is the study design; a cross-sectional study design was employed to explore potential correlates of movement behaviors, based on the research conditions. Consequently, the study cannot establish cause-and-effect relationships. Future studies must address these limitations to develop better and more generalizable research findings.

## 5. Conclusions

The majority (97%) of schoolchildren from low- and middle-income families did not meet the 24-h movement guidelines, raising serious concerns about their movement behaviors. With regard to the rates of meeting the MVPA, ST, and SLP guidelines, large variations were confirmed, which should be addressed in future research. Residential areas and grades are two important factors that showed correlations with healthy behaviors; thus, investigating these links in more detail may permit more specific interventions to be implemented to improve health outcomes among schoolchildren.

## Figures and Tables

**Table 1:** Characteristics of the participants in this study (*n* = 2614).

Category	N	%
**Sex**		
Boys	1285	49.2
Girls	1329	50.8
**Living Areas**		
Urban	1351	51.7
Rural	1263	48.3
**Grade**		
4–6th	877	33.6
7–9th	807	30.8
10–12th	930	35.6
**Family Composition**		
Single	1355	51.8
Two or more	1259	48.2
**Family Income**		
Low	1056	40.4
Low-middle	1558	59.6
**Parental Educational Level**		
Under college or university	2372	90.7
College or university or higher	242	9.3
**Weight Status**		
Normal	2022	77.4
Overweight	414	15.8
Obesity	178	6.8

**Table 2:** Differences in the prevalence of meeting the MVPA, ST, SLP, and 24-h guidelines.

Category	MVPA		*p*	ST		*p*	SLP		*p*	24-h Guidelines		*p*
	*n*	%		*n*	%		*n*	%		*n*	%	
**Total**	260	9.9		1705	65.2		973	37.2		76	2.9	
**Sex**												
Boys	149	11.6	0.006	800	62.3	0.002	487	37.9	0.482	41	3.2	0.397
Girls	111	8.4		905	68.1		486	36.6		35	2.6	
**Living Areas**												
Urban	167	12.4	0.000	928	68.7	0.000	473	35.0	0.016	54	4.0	0.001
Rural	93	7.4		777	61.5		500	39.6		22	1.7	
**Grade**												
4–6th	112	12.8	0.001	545	62.1	0.000	676	77.1	0.000	52	5.9	0.000
7–9th	77	9.5		489	60.6		205	25.4		18	2.2	
10–12th	71	7.6		671	72.2		92	9.9		6	0.6	
**Family Composition**												
Single	146	10.8	0.142	888	65.5	0.730	466	34.4	0.002	37	2.7	0.577
Two or more	114	9.1		817	64.9		507	40.3		39	3.1	
**Family Income**												
Low	113	10.7	0.289	671	63.5	0.137	391	37.0	0.864	30	2.8	0.868
Low-middle	147	9.4		1034	66.4		582	37.4		46	3.0	
**Parental Educational Level**												
Under college or university	236	9.9	0.987	1516	63.9	0.000	907	38.2	0.001	65	2.7	0.111
College or university or higher	24	9.9		189	78.1		66	27.3		11	4.5	
**Weight Status**												
Normal	193	9.5	0.137	1342	66.4	0.700	724	35.8	0.009	57	2.8	0.698
Overweight	52	12.6		256	61.8		167	40.3		12	2.9	
Obesity	15	8.4		107	60.1		82	46.1		7	3.9	

MVPA: moderate to vigorous physical activity; ST: screen time; SLP: sleep.

**Table 3:** Correlates of the prevalence of meeting the MVPA, ST, SLP, and 24-h guidelines.

Category	MVPA		ST		SLP		24-h Guidelines	
	aOR	95%CI	aOR	95%CI	aOR	95%CI	aOR	95%CI
**Sex**								
Boy	1.5	1.1–1.9	Reference		Non-significant		Non-significant	
Girl	Reference		1.3	1.1–1.5				
**Living Areas**								
Urban	1.8	1.4–2.4	1.4	1.2–1.7	Reference		2.5	1.5–4.1
Rural	Reference		Reference		1.3	1.1–1.6	Reference	
**Grade**								
4–6th	1.7	1.3–2.4	Reference		31.2	23.8–40.8	9.3	4.0–21.9
7–9th	1.1	0.8–1.6	0.9	0.7–1.1	3.3	2.5–4.3	2.8	1.1–7.3
10–12th	Reference		1.6	1.3–2.0	Reference		Reference	
**Family Composition**								
Single	Non-significant		Non-significant		Non-significant		Non-significant	
Two or more								
**Family Income**								
Low	Non-significant		Non-significant		Non-significant		Non-significant	
Low-middle								
**Parental Educational Level**								
Under college or university	Non-significant		Reference		Non-significant		Non-significant	
College or university or higher			1.7	1.3–2.4				
**Weight Status**								
Normal	Non-significant		Non-significant		Non-significant		Non-significant	
Overweight								
Obesity								

aOR: adjusted odd ratios; CI: confidence interval.
